# Trend and Spatial Distribution of Drug-Resistant Tuberculosis in Liberty-Deprived Populations in the State of Paraná, Brazil

**DOI:** 10.3390/tropicalmed7100266

**Published:** 2022-09-25

**Authors:** Márcio Souza dos Santos, Thaís Zamboni Berra, Alessandro Rolim Scholze, Felipe Mendes Delpino, Antônio Carlos Vieira Ramos, Yan Mathias Alves, Fernanda Bruzadelli Paulino da Costa, Juliane de Almeida Crispim, Clóvis Luciano Giacomet, Heriederson Sávio Dias Moura, Titilade Kehinde Ayandeyi Teibo, Ana Carolina Silva Peres, Giselle Lima de Freitas, Flávia Meneguetti Pieri, Ricardo Alexandre Arcêncio

**Affiliations:** 1 Department of Maternal-Infant and Public Health Nursing, Ribeirão Preto College of Nursing, University of São Paulo, Ribeirão Preto 05403-000, SP, Brazil; 2 Department of Nursing, Federal University of Minas Gerais, Belo Horizonte 30130-100, MG, Brazil; 3 Department of Nursing, State University of Londrina, Londrina 86039-440, PR, Brazil

**Keywords:** tuberculosis, time series studies, spatial analysis, prisoners, public health

## Abstract

(1) Background: Tuberculosis remains a public health problem in the world. This study evaluated the temporal trends, distribution, and spatial associations of drug-resistant tuberculosis in liberty-deprived populations in the state of Paraná, Brazil. (2) Methods: An ecological study was developed using all cases of drug-resistant tuberculosis in penal establishments reported by the Brazilian Notifiable Diseases Information System between 2008 and 2018. For the time trend, the Prais–Winsten method was used. To verify the spatial association, the Getis–Ord Gi* technique was used. (3) Results: During the study period, 653 cases of tuberculosis were reported in the studied population, of which 98 (15%) were drug-resistant. Prais–Winsten autoregression identified an increasing trend, with APC = 15.08% (95% CI: 0.02–0.09) from 2008 to 2018; when analyzed from 2012 to 2018, the trend increased even more, with APC = 23.31% (95% CI: 0.01–0.16). Hotspots were also noted in the north, east, and west macro-regions of the state. (4) Conclusions: The presence of drug-resistant tuberculosis, as well as an increasing trend of these cases, was evidenced in all regions of the state among liberty-deprived populations,. The spatial analysis revealed priority areas for drug-resistant tuberculosis in penal establishments.

## 1. Introduction

Tuberculosis (TB) is a public health problem on a global scale and mainly affects developing countries with vulnerability and poverty [1]. The occurrence and transmission of the disease are associated with the population’s living conditions and are higher in places of high population density, absence of or precarious basic sanitation and housing infrastructure, poor nutrition, use of illicit drugs, and difficulty in accessing health services [2]. Thus, the populations most vulnerable to TB are people deprived of their liberty, the homeless, immigrants, health professionals, and people living with HIV [2].

It is noteworthy that “liberty-deprived population” refers to the study population (prisoners), and “prison” refers to the place where people who have been convicted of an offense receive punishment. Furthermore, in the present study, the term “penal establishments” was also used, since in Brazil and consequently in the state of Paraná, a person deprived of liberty can be sent to a public prison that is intended for the collection of provisional prisoners or a penitentiary for those sentenced to prison, whose term is to be served in a closed regime: an agricultural and industrial colony intended for prisoners. Penal establishments are observation centers where general and criminological exams are carried out with a house/hospital for custody and psychiatric treatment, which aims to ensure the safe custody of the inmate; in this way, this entire population is captured.

Regarding care for TB patients deprived of liberty, in Brazil, it is determined that they are to be isolated for a period of 15 days after starting treatment in the following cases: when diagnosed with TB at the time of admission to the penal establishment, upon suspected or confirmed resistance, and when treatment failure occurs. In this way, for there to be a cure, supervised treatment must take place daily at the nursing station and the type of treatment must follow that recommended in the primary care notebooks of the Ministry of Health [3].

Health care for TB is not limited to diagnosis and should be focused on comprehensive care and directed to the individual affected by the disease; that is, care begins from early diagnosis to the completion of treatment for a complete “cure” [1]. This is because TB is a curable, preventable disease, and approximately 85% of people who develop it can be treated and cured within 6 months of diagnosis [1].

Even in the face of this recommendation, active TB cases remain within penal establishments, a factor that demonstrates how serious the scenario experienced by inmates is. The lack of early diagnosis and a correct health surveillance system in the penitentiary contributes to the development of resistance to drugs used for the treatment of TB [4,5].

In addition to aspects related to vulnerability, resistance to treatment is a threat for the global epidemiology of TB and triggers worse prognoses related to increased treatment time and lower cure rate [6]. Drug-resistant tuberculosis (DR-TB) is defined by resistance to any drug used to treat TB, mainly rifampicin and isoniazid. This resistance is confirmed through a sensitivity test; the drugs tested are streptomycin, isoniazid, rifampicin, ethambutol, and pyrazinamide [3].

DR-TB is more frequent among socially vulnerable groups, including liberty-deprived populations, which are 28 times more likely to develop the disease when compared to the general population [3,7]. Cases in liberty-deprived populations exceed cases of TB-HIV co-infection, representing the highest proportion of new cases among the populations most vulnerable to TB illness, with an increasing rate: 6.4% in 2010 to 11.1% in 2019 [8].

Worldwide, these are 3.3% of new and medicated cases, and 18% of these known cases are drug-resistant. According to the World Health Organization, 465,000 new TB cases were resistant to rifampicin, of which 78% progressed to multidrug-resistant TB, in 2019. The countries with the highest burdens of the disease are India (27%), China (14%), and Russia (8%). Brazil is not among the countries with the highest burden of drug-resistant TB; however, approximately 2% of cases in Brazil are resistant to rifampicin and less than 3% are multidrug-resistant [1].

This scenario proves the need to develop studies that can support decision-making for controlling and combating the disease, which is necessary for achieving sub-item 3.3 of the 2030 sustainable development agenda: the ending of epidemics, including TB [9,10].

As TB is curable, understanding the trend and spatial distribution of DR-TB in liberty-deprived populations can contribute to opportunities for improvement of work processes, as well as allow for new strategies to control and combat the disease. Thus, the aim of this study was to assess the trend and distribution of DR-TB cases in liberty-deprived populations in the state of Paraná, Brazil.

## 2. Materials and Methods

### 2.1. Study Scenario

**Figure 1:**
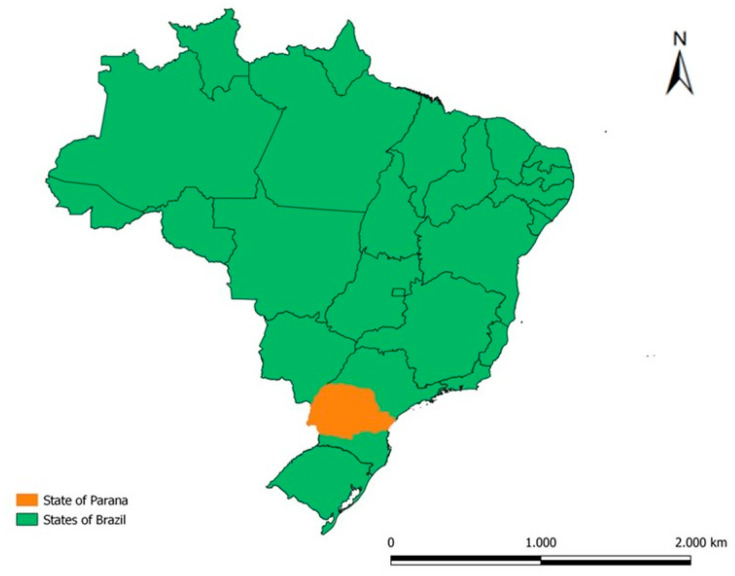
Geographic location of the state of Paraná in Brazil. Legend: ArcGIS [GIS software]. Version 10.0. Redlands, CA, USA, EUA: Environmental Systems Research Institute, Inc., 2010.

This ecological study was carried out in the state of Paraná (Figure 1), which has an area of approximately 199,298,981 km^2^ and a population density of 52.40 inhabitants per km^2^. It had an estimated population of 11,597,484 inhabitants in 2021, 85.3% of whom lived in urban areas [11,12]. Figure 1 illustrates the location of the state.

In the state of Paraná, when TB is suspected, rapid molecular test cultures and sensitivity tests (tests for resistance to streptomycin, isoniazid, rifampicin, ethambutol, and pyrazinamide) are performed to confirm the diagnosis and initiate treatment. From 2013 onwards, the GeneXpert MTB-RIF was approved for incorporation into the Unified Health System; however, it should be noted that this is considered an auxiliary test: it does not replace smear microscopy, which is still used for diagnosis and disease follow-up [13].

When TB is confirmed, the case is reported to SINAN and monitored by the health team and by the State Program for Tuberculosis Control of Paraná. When drug resistance is identified, the case is no longer treated in primary care and is followed up in tertiary care or tuberculosis reference units. The case is also monitored by the Tuberculosis Special Treatments Information System, which in addition to providing case management, carries out medication management by dispensing, requesting, receiving, transferring, and controlling stock.

Regarding the structure, the prison system is divided into nine regions in the state and consists of 55 penal establishments. In Brazil, in 2019, the total liberty-deprived population exceeded 748,000 people, with 3.98% of this total concentrated in Paraná, comprising about 29,831 people [14].

### 2.2. Population, Source of Information, and Selection Criteria

The study population consisted of all susceptible TB cases and bacteriologically confirmed DR-TB cases that were reported among liberty-deprived populations in the Notifiable Diseases Information System (SINAN) between the years of 2008 to 2018.

In Brazil, since the enactment of Law N°6259 on 30 October 1975, all health professionals and those responsible for organizations of public and private health and teaching establishments are required to fill out a suspected case notification form. The form enables reporting of known or suspected cases of diseases that are subject to compulsory notification; after notification, the data are fed via virtual form into the SINAN platform [15].

In order to access these databases, consent was requested from the coordinator of the State TB Control Program. The data were made available by the coordinator of the State TB Control Program of the Department of Epidemiological Surveillance of the State Health Department of Paraná. Duplicate cases were excluded, and the most current record was considered.

### 2.3. Data Analysis

For the georeferencing stage, all cases were considered according to the municipalities of the penal establishment. Notifications with incomplete addresses were excluded as the geographic coordinates could not be ascertained.

First, calculation of the monthly incidence rate of DR-TB was performed considering the direct standardization method [16], which is important for comparing health indicators on a more realistic basis. For this process, the Microsoft Office Professional Plus 2016 program was used, through Excel.

The Prais–Winsten autoregression method was performed using the STATA version 14 software to classify the temporal trend as increasing, decreasing, or stationary. For cases in which the time trend was classified as increasing or decreasing, the percentage of annual variation (APC) was calculated [17]:

𝐴𝑃𝐶 = [−1 + 10𝑏] × 100%

𝐼𝐶95% = [−1 + 10𝑏1𝑚𝑖𝑛] × 100%; [−1 + 10𝑏1𝑚𝑎𝑥] × 100%

For modeling and enabling the prediction of the time series, we used the Box–Jenkins methodology (1976) or the Integrated Autoregressive Moving Averages Model (ARIMA) [18], which is quite flexible and allows adaptations and adjustments in its parameters, carried out via RStudio software [19]. The time series was conducted for two periods: from 2008 to 2018, which comprises the study period, and from 2012 to 2018, justified by the increase in notifications in this period.

In order to analyze the spatial distribution, we started with the geocoding of the cases. At this stage, the coordinates of the Universal Transverse Mercator System (UTM) were determined from the municipality of the penal establishment using the free software Google Earth™ Version 7.15 (Google LLC, Menlo Park, CA, USA). The cartographic base of the municipalities was obtained from the website of the Brazilian Institute of Geography and Statistics (IBGE) free of charge. For the elaboration of the georeferencing maps of the cases, the ArcGis software version 10.5 (EUA: Environmental Systems Research Institute, Redlands, CA, USA) was used.

Note that the municipality in which the penal establishment was located was considered as the ecological analysis unit, since it was not possible to identify the address of the penal establishment in the available database due to data protection law [20].

To verify spatial association, the Getis-Ord Gi* technique was used, which consists of a local association indicator that considers the values for each location—in this case, municipality of the state—from a neighborhood matrix. In this analysis, a z-score was generated for statistically significant municipalities: the higher the z-score, the more intense the clustering of high values (Hotspot). For negative z-scores, the logic is the same; that is, the lower the z-score, the more intense the clustering of low values (Coldspot) [21].

In addition to the z-score, the *p*-value and significance level (Gi-Bin) are also provided, which identify statistically significant hot and cold spots. Values can vary between ±3 and reflect statistical significance with a 99% confidence level, ±2 with a 95% confidence level, and ±1 with a 90% confidence level, with the value zero corresponding to non-statistically significant areas [21].

To detect spatial and spatio-temporal clusters of DR-TB cases, we used specialized spatial analysis, also known as Scan Statistics, as developed by Kullffa [22]. In considering a non-purely spatial cluster identification in which the distribution is heterogeneous and the clusters are rare in relation to the population, the Poisson model was used with discrete requirements on the geographical position of the events, 999 replications, and the size of the cluster. Exposed populations were stipulated by the Gini coefficient [22].

Cluster detection analyses were performed using SaTScan™ software version 9.2 (https://www.satscan.org/) and thematic maps containing the relative risks (RRs) of the areas identified in the scan analysis were constructed using QGIS software version 3.22 (QGIS, Beaverton, OR, USA). In all tests, the type I error was set at 5% as statistically significant (*p* < 0.05).

### 2.4. Ethical Aspects

The study was authorized by the Paraná State Health Department—SESA and approved by the Ribeirão Preto School of Nursing with the Presentation Certificate for Ethical Assessment (CAAE) No. 31631520.2.0000.5393.

## 3. Results

A total of 653 TB cases were reported in liberty-deprived adults, of which 98 (15%) were DR-TB cases. The proportion of patients with bacteriologically confirmed TB and drug sensitivity test results was 66.32%.

Through the Prais–Winsten auto-regression, in the period between 2008 and 2018, it was possible to observe that the DR-TB rate in the state of Paraná showed an increasing trend, with APC = 15.08% (95% CI: 0.02–0.09), and when analyzed from 2012 to 2018, there was a greater increasing trend in the number of registered DR-TB cases, with APC = 23.31% (95% CI: 0.01–0.16).

**Figure 2:**
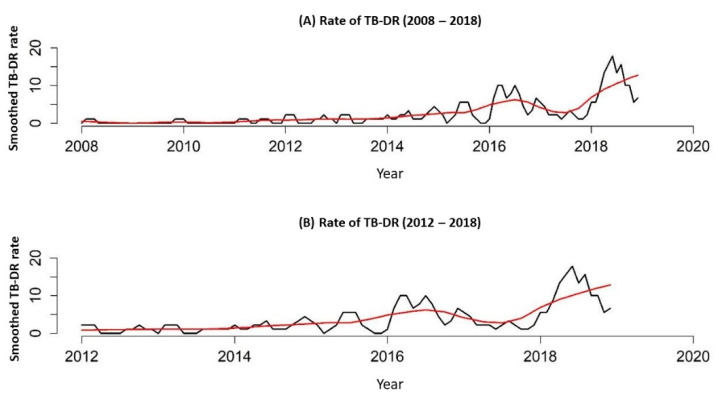
DR-TB rate in the liberty-deprived population of the state of Paraná, Brazil, 2008 to 2018. (**A**), DR-TB rate in the liberty-deprived population of the state of Paraná, Brazil, 2008 to 2018; (**B**) DR-TB rate in the liberty-deprived population of the state of Paraná, Brazil, 2012 to 2018.

In Figure 2, it is possible to see the DR-TB rate/100,000 inhabitants for two periods, 2008 to 2018 and 2012 to 2018, proving the growing trend.

**Figure 3:**
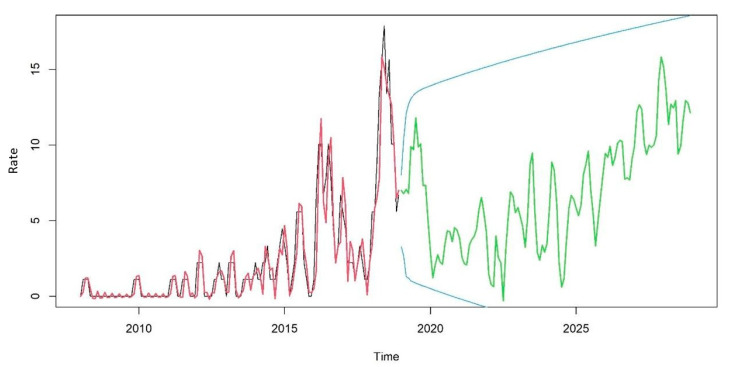
TBDR trend from 2018 to 2028 in the state of Paraná, Brazil.

When calculating the simulated ARIMA, an increasing trend was observed for the coming years that remained within the 95% confidence interval, as shown in Figure 3.

**Figure 4:**
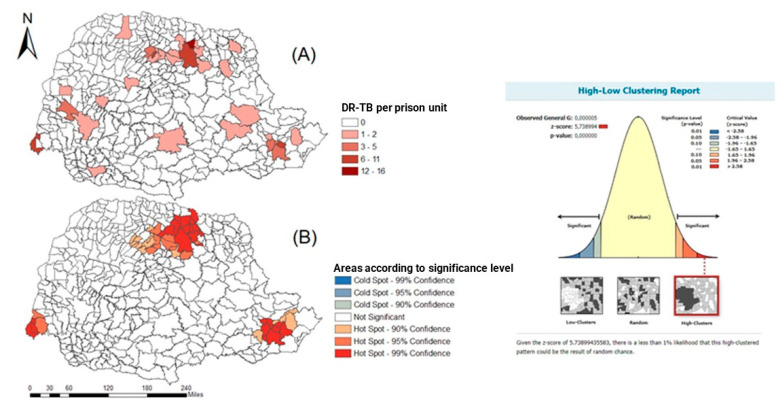
Spatial distribution of DR-TB cases in the liberty-deprived population in the state of Paraná, Brazil, 2008 to 2018. (**A**) DR-TB in the iberty-deprived population is present in all regions of the state, with a greater number of cases in clusters in the northern region. (**B**) hotspots in the north, east, and west of the state, with a statistical significance level above 90% confidence.

The spatial distribution of freedom–DR cases in the private population of the state of Paraná and the freedom sample can be seen in Figure 4, as well as the cases of freedom–DR in the penal establishment of Paraná and spacial association trends for north, east, and west macro-regions.

From Figure 4A, it is possible to see that DR-TB in the iberty-deprived population is present in all regions of the state, with a greater number of cases in clusters in the northern region. Figure 4B shows hotspots in the north, east, and west of the state, with a statistical significance level above 90% confidence.

**Figure 5:**
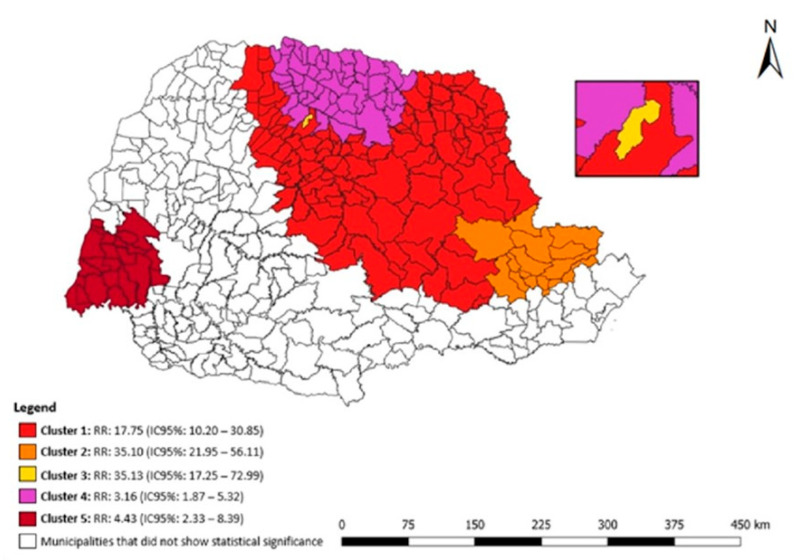
Areas of spatial risk for DR-TB in the liberty-deprived population in the state of Paraná, Brazil, 2008 to 2018.

Figure 5 shows the statistical results of the purely spatial scan for DR-TB in the penal establishment in the state of Paraná. Five clusters of spatial risk were identified: (1) 182 municipalities had an RR: 17.75 (95% CI: 10.20–30.85), (2) 27 municipalities had an RR: 35.10 (95% CI: 21.95–56.11), (3) the municipality of Sarandi had an RR: 35.13 (95% CI: 17.25–72.99), (4) 49 municipalities had an RR: 3.16 (95% CI: 1.87–5.32), (5) 21 municipalities had an RR: 4.43 (95% CI: 2.33–8.39).

## 4. Discussion

The present study analyzed the trend and distribution of DR-TB cases in the iberty-deprived population in the state of Paraná, Brazil. After analyzing the cases, a growing trend towards DRTB was identified in penal establishments in the state of Paraná. As for the territory, it was observed that the highest risk areas were found in the North and Northwest regions of the state.

The irregular use of the drugs for the treatment of TB is closely related to the emergence of DR-TB, with certainty that treatment failure provides such resistance. The care protocols for the treatment of TB in Brazil consist of basic treatment with first-line drugs, namely rifampicin, ethambutol, and isoniazid for at least six months. When replacement with second-line drugs is necessary, treatment time increases, which may cause another challenge, often resulting in abandonment.

Therefore, improvement of techniques and tools to control and combat this disease, which has both treatment and cure, is necessary to achieve sub-item 3.3 of the 2030 agenda for sustainable development, which proposes an end to epidemics, including TB [10]. While there is a National Policy for Comprehensive Health Care for Persons Deprived of Liberty in the Prison System (PNAISP) in Brazil, it is necessary to improve public policies for tracking, treating, and monitoring affected individuals.

This study also points to an important aggravating factor, which is the growing trend of cases of resistance to drugs used in the treatment of TB. This reality is also present in other countries of the world, which introduces the concern that treatment has not been effective, in addition to the possibility of transmission in its resistant form [4,5,23,24,25].

The cure outcome in 2018 for sensitive TB cases was 71.9% and for resistant cases it was 55.7%. Evidently, TB remains a public health problem [8].

Among the populations vulnerable to TB, the liberty-depriced population stands out, whether because of the health conditions or the environment of penal establishments, which are both favorable for the spread of the disease [3]. From this perspective, it is noteworthy that in 2014, prisons in post-Soviet states exceeded the world prevalence estimated by the World Health Organization (WHO) by 16 times [7].

In addition to DR-TB being present in all regions of the state, when calculating ARIMA, it has been noted that this trend will increase for the next three years. This may be related to treatment failure and the fact that penal establishments are considered a structural/environmental risk factor for the spread of TB due to inadequate ventilation, poor nutrition, inadequate health care, overcrowding, and other structural factors that are predetermining factors for the increase in TB incidence [26,27,28].

Other factors that contribute to the permanence of TB, as well as DR-TB, are the health inequities that are present in the community, which demonstrates a need to address social determinants [29].

While Brazil has the National Policy for Comprehensive Health Care for Persons Deprived of Liberty in the Prison System (PNAISP), established by Interministerial Ordinance No. of the Unified Health System for this public, the present study points to a trend towards an increase in DR-TB cases [30].

The PNAISP stresses the importance of implementing access and reception protocols and, for TB, these are an important actions, as they increase the opportunity for early detection and follow-up for adequate treatment, facilitating better monitoring [30].

Concerning the spatial distribution of cases, clusters were observed in the North, East, and West regions with statistical significance. This may be related to penal establishment case report rates. Thus, we highlight the importance of reporting TB/DR-TB cases, as these data allow for the evaluation and reassessment of work processes and behaviors that can contribute to the control of the disease [31].

Given the above, we recommend that penal establishments outline and rethink effective barrier strategies to prevent TB spread, which remains a public health problem. It is noteworthy that, even in the face of difficulties and limitations, it is possible to change the TB scenario, as observed in a study carried out on the prison system of Azerbaijan, in which comprehensive TB control measures and the guarantee of low losses at follow-up resulted in about 78.4% of the liberty-deprived population with DR-TB being cured [32].

In order to work with tuberculosis data and other SINAN-notifiable diseases, one must understand that the SINAN database is subject to improvement but allows for broader perspectives and proposes improvements in the face of novel research. By employing methodological rigor and intensified research, there is an opportunity to fight against diseases, and in particular, TB, which can be treated but has also demonstrated resistance to extant therapies.

The authors recognize the importance of the subject, and encourage and recommend further research to better understand those factors that are associated with drug resistance.

## 5. Conclusions

In view of the above, it is evident that penal establishments contribute to the burden of TB and, consequently, become a favorable environment for the maintenance of the disease, including its drug-resistant form.

Our study emphasizes that DR-TB in the liberty-deprived population constitutes a global public health problem, describes a growing trend in TB for the coming years, and highlights a statistically significant spatial association in three regions of the State of Paraná. To address these issues, health managers need to direct their gaze towards this disease and work to enhance public policies for the screening, treatment, and follow-up of affected individuals.

To facilitate this, further, broader studies that seek to understand the dynamics of TB in the liberty-deprived population should be conducted. Such research can strengthen and stabilize opportunities for improvement within penal establishments, allowing treatment to become more effective and resulting in reduction in the incidence of TB and DR-TB.

## Data Availability

The data presented in this study are available on request from the corresponding author on reasonable request.
